# New approach to simplifying and optimising acute malnutrition treatment in children aged 6–59 months: the OptiMA single-arm proof-of-concept trial in Burkina Faso

**DOI:** 10.1017/S0007114519003258

**Published:** 2020-04-14

**Authors:** Maguy Daures, Kevin Phelan, Mariama Issoufou, Séni Kouanda, Ousmane Sawadogo, Kader Issaley, Cecile Cazes, Benjamin Séri, Bertine Ouaro, Bruno Akpakpo, Vincent Mendiboure, Susan Shepherd, Renaud Becquet

**Affiliations:** 1 University of Bordeaux, Inserm, Bordeaux Population Health Research Center, team IDLIC, UMR 1219, Bordeaux, France; 2 The Alliance for International Medical Action (ALIMA), Paris, France; 3 The Alliance for International Medical Action (ALIMA), Yako, Burkina Faso; 4 Département biomédical et santé publique, Institut de Recherche en Sciences de la Sante, Ouagadougou, Burkina Faso; 5 Association KEOOGO, Ouagadougou, Burkina Faso; 6 The Alliance for International Medical Action (ALIMA), Dakar, Senegal; 7 PACCI research programme, University Hospital of Treichville, Abidjan, Ivory Coast; 8 Ministry of Health, Ouagadougou, Burkina Faso

**Keywords:** Acute malnutrition, Children, West Africa, Mid upper arm circumference

## Abstract

The Optimising treatment for acute MAlnutrition (OptiMA) strategy trains mothers to use mid upper arm circumference (MUAC) bracelets for screening and targets treatment to children with MUAC < 125 mm or oedema with one therapeutic food at a gradually reduced dose. This study seeks to determine whether OptiMA conforms to SPHERE standards (recovery rate > 75 %). A single-arm proof-of-concept trial was conducted in 2017 in Yako district, Burkina Faso including children aged 6–59 months in outpatient health centres with MUAC < 125 mm or oedema. Outcomes were stratified by MUAC category at admission. Multivariate survival analysis was carried out to identify variables predictive of recovery. Among 4958 children included, 824 (16·6 %) were admitted with MUAC < 115 mm or oedema, 1070 (21·6 %) with MUAC 115–119 mm and 3064 (61·8 %) with MUAC 120–124 mm. The new dosage was correctly implemented at all visits for 75·9 % of children. Global recovery was 86·3 (95 % CI 85·4, 87·2) % and 70·5 (95 % CI 67·5, 73·5) % for children admitted with MUAC < 115 mm or oedema. Average therapeutic food consumption was 60·8 sachets per child treated. Recovery was positively associated with mothers trained to use MUAC prior to child’s admission (adjusted hazard ratio 1·09; 95 % CI 1·01, 1·19). OptiMA was successfully implemented at the scale of an entire district under ‘real-life’ conditions. Programme outcomes exceeded SPHERE standards, but further study is needed to determine if increasing therapeutic food dosages for the most severely malnourished will improve recovery.

Acute malnutrition is a major public health problem, affecting 49·5 million children worldwide each year and contributing to nearly half of all annual childhood deaths^(1,2)^. One quarter of all acutely malnourished children are in Africa with the largest number, 5·1 million, in West Africa^(2)^. In this region, the burden of acute malnutrition overlaps with the highest rates of child mortality^(2)^. Progress in reducing acute malnutrition needs to accelerate in the region if it is to meet WHO Global Nutrition Targets by 2025^(3)^.

Although acute malnutrition is a continuum condition, it is arbitrarily divided into moderate (MAM) and severe (SAM) categories defined by mid upper arm circumference (MUAC) or weight-for-height *Z*-score (WHZ). MAM (MUAC 115–124 mm or WHZ between –2 and –3) and SAM (MUAC < 115 mm or WHZ <–3 or oedema) are managed separately, with programmes overseen by different UN agencies, and using different protocols and products (ready-to-use supplementary food or fortified-blended flours for MAM and ready-to-use therapeutic food (RUTF) for SAM). Such separation complicates delivery of care, contributes to low coverage and creates confusion among caregivers^(4)^.

Confusion is further exacerbated by a complicated case definition using MUAC and WHZ to determine programme eligibility, even though WHZ does not offer a clear advantage over MUAC for identifying children at near-term risk of death^(5)^. MUAC-only programming is expanding as evidence accumulates that weight gain and MUAC gain track each other and that the trajectory of weight and MUAC gain is maximal during the first 2–3 weeks of treatment^(6–8)^. MUAC is therefore becoming a stand-alone practical tool for all phases of nutrition programming: screening, admission, monitoring recovery and determining discharge.

In addition to the tangle of agencies and case definitions, SAM and MAM programmes are chronically underfunded with only 25 % of SAM cases treated globally in 2016 and 16 % of MAM cases reached by the World Food Program in 2017^(9,10)^. An optimised allocation of resources is therefore needed. A programme integrating SAM and MAM treatment in a single MUAC-based protocol using only RUTF showed the cost of RUTF for integrated treatment to be less than half that for standard SAM management^(11)^. Other studies found that identification and treatment of children earlier in the wasting process led to fewer hospitalisations and that inpatient care was shown to be twice as costly as outpatient SAM management^(12,13)^. Furthermore, the treatment of MAM has been shown to be cost-effective in reducing mortality risk by more than 10 %^(14)^.

We piloted a new MUAC-based and oedema approach for treating acute malnutrition in Burkina Faso with a single-arm proof-of-concept trial called Optimising treatment for acute MAlnutrition (OptiMA). OptiMA made three strategic changes to the current Burkina Faso National Nutrition protocol^(15)^: screening for acute malnutrition was task-shifted to caretakers via community-based training in the use of MUAC bracelets; the case definition for acute malnutrition was modified to MUAC < 125 mm and/or oedema and only one product was used for treatment (RUTF) at a gradually reduced dose based on a child’s weight and MUAC status. We hypothesised that the OptiMA protocol was at least as effective as the national nutrition protocol in terms of children’s recovery rate and other standard programme indicators when compared against international SPHERE standards^(16)^.

## Methods

### Location, period and study population

This single-arm proof-of-concept trial was conducted in Burkina Faso’s Yako Health District. In 2016, Yako had a general population of approximately 415 000, with the under-five population estimated at 82 000, and was served by fifty-four health centres plus a district hospital. Each health centre was staffed by a nurse who routinely provided acute malnutrition treatment. Prevalence of MAM and SAM by WHZ just prior to implementation of OptiMA was 8·2 % and 1·9 %, respectively^(17)^. Health centre staff were trained on the OptiMA protocol in November and December 2016. Inclusions for the study occurred from January through December 2017, and follow-up of study participants ended in March 2018. Children were considered eligible for enrollment if they were aged 6–59 months with a MUAC < 125 mm or bipedal oedema and presented to any of the fifty-four outpatient clinics in Yako District.

### Study protocol

#### *Screening for* acute malnutrition

A mass community-based campaign was conducted from June to August 2016 to train mothers and caretakers throughout Yako District to use MUAC bracelets and check for oedema in order to screen their own children for malnutrition. Following the mass campaign, family MUAC trainings became a routine activity at all fifty-four health centres and continued to the end of the study.

#### Admission/discharge criteria, therapeutic feeding products and dosage

Table 1 summarises the differences between the current national protocol and OptiMA, which was implemented in all of the Yako’s fifty-four health centres for the duration of the study. Children presenting with MUAC ≥ 125 mm and WHZ < −3 were treated in a ‘non-MUAC eligible’ arm according to the current national protocol.

**Table 1.** Admissions and discharge criteria, treatment products and calculation of dosage in the Burkina Faso national and Optimising treatment for acute MAlnutrition (OptiMA) protocols*

	National protocol		OptiMA protocol		
	SAM	MAM	Acute malnutrition		
Admission	MUAC < 115 mm or WHZ < −3 or oedema	MUAC ≥ 115 mm and MUAC < 125 mm or −3 < WHZ < −2	MUAC < 115 mm or oedema	115 ≤ MUAC ≤ 120 mm	120 ≤ MUAC < 125 mm
Treatment product	RUTF 150–200 kcal/kg per d	Super cereal plus 200 g/d or RUSF one 92 g sachet/d	RUTF 175 kcal/kg per d	RUTF 125 kcal/kg per d	RUTF 75 kcal/kg per d
Calculation of dosage	According to the weight	Fixed amount, regardless of weight or MUAC status	According to MUAC status and weight		
Discharge criteria	MUAC ≥ 125 mm for two consecutive visits or WHZ ≥ −2 for two consecutive visits		MUAC ≥ 125 mm for two consecutive weeks		
	No oedema minimum 2 weeks	After recovery from SAM: discharge after 3 months without losing weight	No oedema for minimum 2 weeks		
	Minimum 4 weeks in programme		Minimum 4 weeks in programme		
	Good clinical health		Good clinical health		

SAM, severe acute malnutrition; MAM, moderate acute malnutrition; MUAC, mid upper arm circumference; WHZ, weight-for-height *Z*-score; RUTF, ready-to-use therapeutic food; RUSF, ready-to-use supplementary food.

* To convert kcal to kJ, multiply by 4·184.

In contrast to the weight-based RUTF ration in the national SAM programme, which is fixed at 150–200 kcal/kg per d (628–837 kJ/kg per d) for the course of treatment, the OptiMA RUTF ration was calibrated to the child’s degree of wasting based on the combination of MUAC status and weight. Thus, more nutritional support was given to the most severely malnourished and then gradually reduced as the child’s MUAC increased. Children with MUAC < 115 mm or oedema received 175 kcal/kg per d (732 kJ/kg per d) of RUTF. Children with MUAC 115–119 mm, either at admission or during the course of treatment, received 125 kcal/kg per d (523 kJ/kg per d) of RUTF, and children with MUAC ≥ 120 mm received 75 kcal/kg per d (314 kJ/kg per d) of RUTF (with a minimum of one sachet/d) until discharge from the programme.

#### Systematic medical treatment

All children underwent malaria rapid testing upon admission and at any point during their participation if clinical signs of malaria were detected. All children with a positive malaria rapid diagnostic test were prescribed an artemisinin-combination treatment. Amoxicillin 90 mg/kg per d for 7 d was prescribed for all children with MUAC < 120 mm or oedema. Albendazole was given to children if they had no deworming in the previous 4 months.

### Defaulter follow-up

Community health workers traced children who were classified as defaulters to their caretakers’ homes and completed a standardised form recording vital status, MUAC measure and reasons for defaulting.

### Primary and secondary outcomes

The primary outcome was recovery defined as the proportion of children achieving a MUAC ≥ 125 mm and absence of oedema for two consecutive weeks, in good clinical health, with a minimum programme stay of 4 weeks. Secondary outcomes were RUTF consumption and other standard programme indicators: death (occurring after programme admission and prior to recovery), default (being absent for three consecutive visits), non-response (failure to achieve recovery after 12 weeks in the programme) and hospitalisations (children referred for inpatient care at admission or at any point during the course of treatment).

### Data collection procedures and monitoring

One of the five supervisors visited each health facility bimonthly to ensure protocol adherence and correct completion of patient records. There was no increase in clinical staff to manage the nutrition programme.

Data were collected using the national programme individual outpatient records (modified to include information on family MUAC-training). These forms were entered into an anonymised Access database. Data monitoring was conducted from completed archived patient records and included searching for each child’s anonymous unique identifier in health centres in order to ensure that all children enrolled were in the database.

Socio-demographic information on the caretaker and child, mode of referral, distance to the health centre, caretaker MUAC-training prior to admission, Amoxicillin prescription and results of malaria rapid diagnostic test were collected at the first visit. The child’s weight, MUAC, temperature, clinical symptoms and amount of RUTF ration were recorded at each weekly visit. Children’s length was measured at admission and once a month thereafter. Weight was measured to the nearest 100 g with a Salter scale, and length was measured to the nearest 0·5 cm on a height board with the child in a supine position (or standing if taller than 85 cm). MUAC was measured to the nearest mm with a MUAC bracelet demarcated in 1 mm increments. At each visit, supervisors ensured that scales were correctly calibrated and MUAC bracelets and height boards were in good condition.

### Data analysis

Data analysis was performed using RStudio software (RStudio, Inc.).

WHZ, weight-for-age *Z*-score and height-for-age *Z*-score (HAZ) were calculated using WHO 2006 growth standards.

In order to evaluate adherence to the new OptiMA dosage tables, the difference between weekly RUTF ration provided to the child and the theoretical OptiMA ration as calculated by the new tables were described.

Children who were alive but erroneously classified as recovered (after a single MUAC measurement ≥ 125 mm) or defaulter (prior to three consecutive weeks of absence) or non-respondent (before 12 weeks of participation without achieving discharge criteria), and who did not correspond to another category of exit, were reclassified as ‘Alive, unconfirmed status’.

Programme outcomes were described overall and stratified by MUAC category at admission with their 95 % CI and considered at least as effective as the international SPHERE standards if the lower limit of the 95 % CI was greater than or equal to the reference value.

Multivariable survival analysis to identify variables predictive of recovery was run using a shared frailty model^(18)^ (extension of the Cox proportional hazard model), with a random effect on health centres, and by assuming a Weibull distribution for the baseline hazard function. A univariate analysis by fitting a separate model for each covariate was done, variables having a *P*-value ≤ 0·2 were entered into multivariate analysis. *P*-value < 0·05 was considered as statistically significant in the final model.

To investigate the contribution of factors that explain the recovery (i.e. the proportion of recovered children explained by each variable of interest), adjusted attributable fraction (AF) of risks was estimated based on the final multivariate model. The AF for each factor was obtained by the difference between the predicted number of recovered children in a simulated dataset where exposure to each variable was removed and the real number of recovered children in OptiMA database^(19)^. The adjusted AF were estimated in relation to the recovery at the median length of stay in the programme. We computed 95 % CI using bootstrapping^(19)^.

### Ethics

The present study was approved by the Ethics Committee for Health Research (2016-6-067) and Technical Review Committee for Clinical Trial Authorizations (5003720165EC0000) of Burkina Faso’s Ministry of Health. The trial was registered on clinicaltrials.gov (NCT03027505). Caregivers gave written consent (signature or fingerprint) prior to enrollment for all children included in the study. All data were anonymised when entered into the database, and unique identification numbers were coded. A data safety monitoring board conducted an independent interim analysis, reviewed consolidated results and endorsed completion of the study.

## Results

**Fig. 1.**
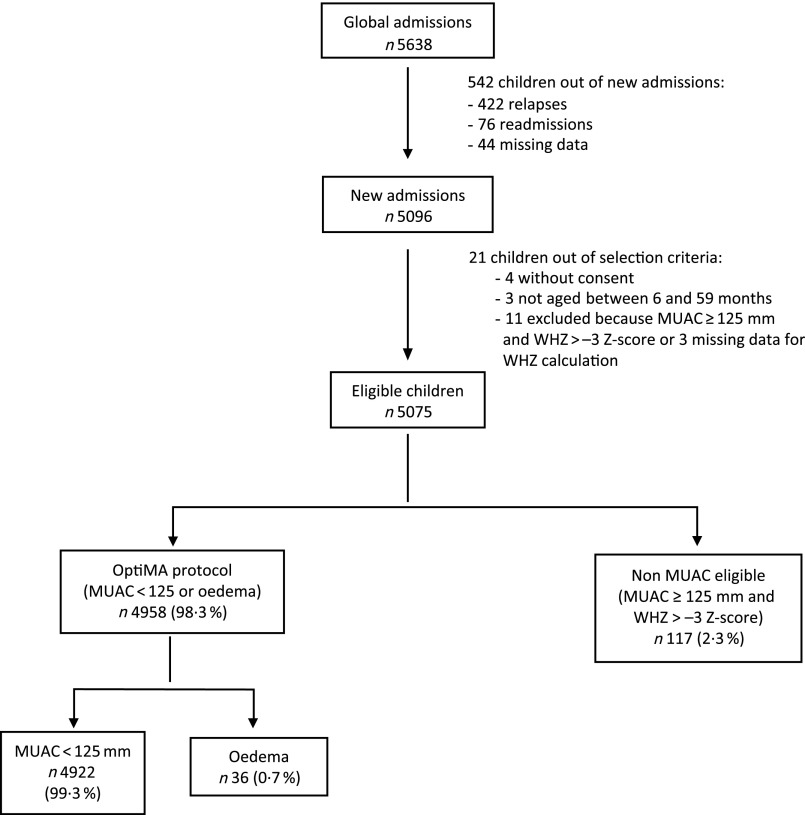
Flow chart of children admitted under the Optimising treatment for acute MAlnutrition (OptiMA) protocol, Yako district, Burkina Faso, 2017. MUAC, mid upper arm circumference; WHZ, weight-for-height *Z*-score.

Of the 5638 children included in the nutrition programme from January to December 2017, 4958 were included for analysis (Fig. 1). Children omitted from analysis were those who relapsed, were readmitted, had missing data, did not meet OptiMA selection criteria or were not MUAC eligible (MUAC ≥ 125 mm and WHZ < –3). Non-MUAC eligible children are described in online Supplementary file 1.

Baseline characteristics of children included under OptiMA are presented in Table 2. A majority were girls (58·4 %) and younger than 24 months (82·2 %). Overall, 0·7 % of children were admitted with oedema, 15·9, 21·6 and 61·8 % with MUAC < 115, 115–119 and 120–124 mm, respectively. Upon admission, 71·0 % of children met both MUAC and WHZ definition for wasting (28·8 % with WHZ <–3, and 42·2 % with WHZ between –3 and –2), while 29·0 % met the MUAC definition only. Stunting was present in 42·0 % of children. The proportion and severity of wasting by WHZ, stunting and underweight were highest among children admitted with MUAC <115 mm. Most caretakers (77·1 %) received MUAC training prior to child’s admission.

**Table 2.** Description of children included in Optimising treatment for acute MAlnutrition (OptiMA) protocol, Yako district, Burkina Faso, 2017 (Numbers and percentages; mean values and standard deviations)

	Overall (*n* 4958)		MUAC <115 mm or oedema (*n* 824)		MUAC 115–119 mm (*n* 1070)		MUAC 120–124 mm (*n* 3064)	
	*n*	%	*n*	%	*n*	%	*n*	%
Child socio-demographics characteristics								
Age (months)								
Mean	14·9		12·7		14·5		15·7	
sd	8·8		8·4		8·5		8·9	
Age category								
<24 months	4077	82·2	728	88·3	901	84·2	2448	79·9
≥24 months	881	17·8	96	11·7	169	15·8	616	20·1
Sex (*n* 4941)								
Male	2057	41·6	326	39·8	421	39·6	1310	42·8
Female	2884	58·4	494	60·2	641	60·4	1749	57·2
Mode of referral (*n* 4845)								
Community agent	1904	39·3	266	33·2	406	38·7	1232	41·2
Mothers screening	628	12·9	154	19·2	158	15·1	316	10·5
Outpatient consultation	2253	46·5	359	44·7	476	45·3	1418	47·4
Inpatient consultation	60	1·2	23	2·9	10	0·9	27	0·9
Distance to the health centre (*n* 4703)								
≥10 km	447	9·5	113	14·4	122	12·0	212	7·3
<10 km	4256	90·5	670	85·6	894	88·0	2692	92·7
Mother’s characteristics at admission								
Mother’s status (*n* 4873)								
Alive	4806	98·6	786	97·4	1038	98·2	2982	99·1
Dead	67	1·4	21	2·6	19	1·8	27	0·9
No. of siblings (*n* 4697)								
0	18	0·4	5	0·7	6	0·6	7	0·2
1–4	2711	57·7	433	55·9	595	59·4	1683	57·6
4–12	1968	41·9	336	43·4	400	40·0	1232	42·2
Breast-feeding (*n* 4512)								
Yes	3820	84·7	652	87·7	808	84·3	2360	84·0
No	692	15·3	91	12·3	151	15·7	450	16·0
Years of schooling (*n* 4442)								
No	3941	88·7	672	90·8	855	89·6	2414	87·8
≥1 year	501	11·3	68	9·2	99	10·4	334	12·1
MUAC training (*n* 4844)								
Yes	3733	77·1	614	76·2	772	74·2	2347	78·3
No	1111	22·9	192	23·8	268	25·8	651	21·7
Anthropometric characteristics								
MUAC at admission (mm) (*n* 4922)								
Mean	118·7		109·5		116·8		121·7	
sd	5·2		5·7		1·5		1·6	
WHZ (*n* 4777)								
Mean	−2·5		−3·0		−2·6		−2·3	
sd	0·9		1·1		0·9		0·9	
WHZ categories								
<−3	1377	28·8	423	56·3	357	34·6	597	19·9
−3 and −2	2017	42·2	239	31·8	450	43·6	1328	44·3
≥−2	1383	29·0	89	11·9	224	21·7	1070	35·7
HAZ (*n* 4760)								
Mean	−1·7		−2·2		−1·8		−1·6	
sd	1·4		1·3		1·4		1·3	
HAZ categories								
<−3	848	17·8	205	27·1	217	21·2	426	14·3
−3 and −2	1150	24·2	216	28·6	253	24·8	681	22·8
≥−2	2762	58·0	335	44·3	552	54·0	1875	62·9
WAZ (*n* 4772)								
Mean	−2·8		−3·4		−2·9		−2·5	
sd	0·9		0·9		0·8		0·8	
WAZ categories								
<−3	1858	38·9	496	68·7	494	48·1	868	28·7
−3 and −2	2008	42·1	192	26·6	406	39·5	1410	46·7
≥−2	906	19·0	34	4·7	128	12·4	744	24·6
Malaria data								
Malaria RDT (*n* 4293)								
Positive	1103	25·7	192	26·5	275	29·4	636	24·1
Negative	3190	74·3	532	73·5	659	70·6	1999	75·9
Received SMC (*n* 4907)								
Included out of SMC campaign	3550	72·3	615	75·4	785	74·3	2150	70·8
SMC campaign eligible and received	305	6·2	41	5·0	53	5·0	211	6·9
SMC campaign eligible and not received	1052	21·5	160	19·6	218	20·6	674	22·2

MUAC, mid upper arm circumference; WHZ, weight-for-height *Z*-score; HAZ, height-for-age *Z*-score; WAZ, weight-for-age *Z*-score; RDT, rapid diagnostic tests; SMC, seasonal malaria chemoprevention during campaign period from July to October 2017.

**Fig. 2.**
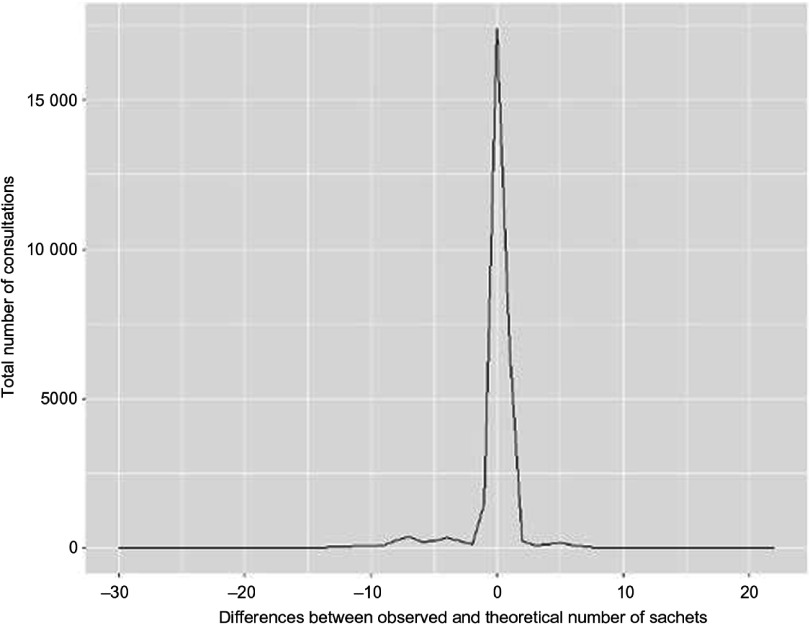
Distribution of the differences by consultation between the number of ready-to-use therapeutic food sachets given to children and the theoretical dosage under Optimising treatment for acute MAlnutrition protocol, Yako district, Burkina Faso, 2017.

Fig. 2 shows the difference by visit between the number of RUTF sachets given to children and the theoretical dosage per OptiMA protocol. The new dosage regimen was correctly implemented at all visits for 75·9 % of children. For children who received a dosage error, the distribution shows that for most of the visits, the mistake was within two sachets. Mothers who notified health workers of a planned absence received 2 or 3 weeks of RUTF ration at one visit, which may explain the larger variations.

Overall recovery exceeded the SPHERE standard for both SAM and MAM programmes: 86·3 % (95 % CI 85·4, 87·2) (Table 3). Recovery was lowest among children who were most malnourished at admission, with 70·4 % (95 % CI 67·5, 73·5) of the children admitted with MUAC < 115 mm or oedema recovering. There were 243 (4·9 %) children who were alive at discharge but wrongly classified by nurses, with 122 (50·0 %) of these children erroneously classified as recovered with one MUAC measure ≥ 125 mm instead of two. Mortality rate was low (0·4 %; 95 % CI 0·0, 1·3), while defaults, non-response and transfers represented 4·7 % (95 % CI 3·8, 5·6), 3·4 % (95 % CI 2·5, 4·3) and 0·2 % (95 % CI 0·0, 1·1), respectively.

**Table 3.** Programme outcome globally and by mid upper arm circumference (MUAC) category at admission among children treated by Optimising treatment for acute MAlnutrition (OptiMA) protocol, Yako district, Burkina Faso, 2017 (Numbers and percentages; 95 % confidence intervals; mean values and standard deviations)

	National standard (%)	SPHERE standard (%)				MUAC category at admission								
			Overall (*n* 4958)			<115 mm or oedema (*n* 824)			115–119 mm (*n* 1070)			120–124 mm (*n* 3064)		
			*n*	%	95 % CI	*n*	%	95 % CI	*n*	%	95 % CI	*n*	%	95 % CI
Exit status														
Recovered	75	75	4279	86·3	85·4, 87·2	580	70·4	67·5, 73·5	900	84·1	82·1, 86·2	2799	91·4	90·4, 92·2
Deceased	3	10	22	0·4	0·0, 1·3	12	1·5	0·0, 4·5	6	0·6	0·0, 2·6	4	0·1	0·0, 1·0
Defaulted	15	15	233	4·7	3·8, 5·6	77	9·3	6·4, 12·4	59	5·5	3·6, 7·6	97	3·2	2·2, 4·1
Non-responders			170	3·4	2·5, 4·3	87	10·6	7·6, 13·6	46	4·3	2·3, 6·4	37	1·2	0·2, 2·1
Transferred			11	0·2	0·0, 1·1	4	0·5	0·0, 3·5	1	0·1	0·0, 2·2	6	0·2	0·0, 1·1
Alive: unconfirmed status			243	4·9	4·0, 5·8	64	7·8	4·8, 10·8	58	5·4	0·3, 7·5	121	3·9	3·1, 4·9
Hospitalisation														
At least one hospitalisation			680	13·7	12·8, 14·7	269	32·6	29·5, 36·0	186	17·4	15·2, 19·7	225	7·3	6·4, 8·2
Hospital mortality			6	0·9	0·0, 4·0	3	1·1	0·0, 6·5	3	1·6	0·0, 7·7	0		
Length of hospital stay (d)														
Mean			8·4			9·2			8·2			7·5		
sd			5			5·7			4·5			4·2		

Table 4 shows programme outcomes stratified by MUAC and WHZ at admission. Children with both MUAC < 115 mm and WHZ <−3 had the lowest recovery (64·3 %, 95 % CI 60·0, 68·9) and highest non-response (12·5 %, 95 % CI 8·3, 17·1) and mortality (1·7 %, 95 % CI 0·0, 6·2).

**Table 4.** Programme outcomes among children treated with Optimising treatment for acute MAlnutrition (OptiMA) protocol stratified by mid upper arm circumference (MUAC) category and weight-for-height *Z*-score (WHZ)* at admission, Yako district, Burkina Faso, 2017 (Numbers and percentages; confidence intervals)

	MUAC <115; WHZ <−3 (*n* 423)			MUAC <115; WHZ −3 and −2 (*n* 239)			MUAC <115; WHZ ≥ −2 (*n* 89)			MUAC 115–124; WHZ < −3 (*n* 954)			MUAC 115–124; WHZ −3 and −2 (*n* 1778)			MUAC 115–124; WHZ ≥ −2 (*n* 1294)		
	*n*	%	95 % CI	*n*	%	95 % CI	*n*	%	95 % CI	*n*	%	95 % CI	*n*	%	95 % CI	*n*	%	95 % CI
Recovery	272	64·3	60·0, 68·9	193	80·8	76·6, 85·7	69	77·5	69·7, 85·5	844	84·9	81·2, 89·4	1592	89·5	88·2, 90·9	1176	90·9	89·5, 92·3
Deceased	7	1·7	0·0, 6·2	2	0·8	0·0, 5·7	0			5	0·8	0·0, 5·2	2	0·1	0·0, 1·4	3	0·2	0·0, 1·7
Defaulted	47	11·1	6·8, 15·7	13	5·4	1·2, 10·4	7	7·9	0·0, 15·9	39	5·4	1·7, 9·8	64	3·6	2·3, 4·9	41	3·2	1·8, 4·6
Non-respondent	53	12·5	8·3, 17·1	17	7·1	2·9, 12·1	11	12·4	4·5, 20·4	18	5·4	1·7, 9·8	41	2·3	1·0, 3·6	21	1·6	2·3, 3·1
Transferred	2	0·5	0·0, 5·1	1	0·4	0·0, 5·4	0			3	0·4	0·0, 4·8	1	0·1	0·0, 1·4	2	0·2	0·0, 1·6
Unconfirmed status	42	9·9	5·7, 14·5	13	5·4	1·2, 10·4	2	2·2	0·0 8·0	45	2·9	0·0, 7·3	78	4·4	3·1, 5·7	51	3·9	2·5, 5·4
At least one hospitalisation	151	35·7	31·2, 40·5	67	28·0	22·6, 33·9	14	15·7	8·9, 23·0	121	12·7	10·7, 14·8	175	9·8	8·5, 11·2	89	6·9	5·6, 8·3

* 181 children had missing data on ‘WHZ category’.

Home visits were conducted for 233 children classified as defaulters: 113 were alive and physically seen (average MUAC was 124, sd 7·1), thirty-eight were declared alive by the caretaker but not physically seen, four were deceased and seventy-eight could not be found. Among the seventy-seven defaulters with a MUAC < 115 mm at admission, the average number of visits before default was 4·7 and their last MUAC measure was 115·1 mm on average (sd 8·2).

Of 170 non-responders, eighty-four (49·4 %) were admitted with MUAC < 115 mm and three (1·8 %) for oedema. The average length of stay and MUAC at last visit for all non-responders and those with MUAC < 115 mm or oedema at admission were 14·6 (sd 3·5) weeks, 120·4 (sd 5·6) mm and 15·3 (sd 3·8) weeks, 119·5 (sd 4·6) mm, respectively.

Of the twenty-two deaths, 72·7 % were younger than 24 months, 59·1 % were girls and 54·5 % had a MUAC < 115 or oedema at admission, while this group accounted for only 16·6 % of all admissions. The average time from admission to death was 18·8 (sd 19·2) d, with six, eight and eight deaths occurring on the day of admission, 1–21 and 22–60 d after admission, respectively. Among these sixteen deaths that occurred after the day of admission, ten children had MUAC < 115 mm, nine were girls and the median age was 14 months (interquartile range 7·7, 26·2)

Among all recovered children and those admitted with MUAC < 115 mm or oedema, length of stay was 5·8 and 8·1 weeks, MUAC gain was 9·8 and 17·5 mm, weight gain was 3·7 and 4·7 g/kg per d and the amount of RUTF provided was 51·0 and 87·4 sachets, respectively (Table 5). The OptiMA dosage table provided a slightly lower than the anticipated average daily energetic intake to the most malnourished at 131 kcal/kg per d (548 kJ/kg per d).

**Table 5.** Average length of stay, weight gain, mid upper arm circumference (MUAC) gain and average ready-to-use therapeutic food (RUTF) consumption in recovered children treated with the Optimising treatment for acute MAlnutrition (OptiMA) protocol, Yako district, Burkina Faso, 2017 (Mean values and standard deviations; medians and interquartile ranges (IQR))

				MUAC at admission								
	Overall (*n* 4279)			<115 and oedema (*n* 580)			115–119 (*n* 900)			120–124 (*n* 2799)		
	Mean		sd	Mean		sd	Mean		sd	Mean		sd
Average time to recovery (week)	5·8		2·5	8·1		3·4	6·5		2·7	5·2		1·9
Average MUAC gain (mm)	9·8		5·1	17·5		5·4	11·2		3·2	7·7		3·7
Average weight gain (g/kg per d)	3·7		2·2	4·7		2·5	3·8		1·9	3·5		2·2
Average no. of RUTF	58·9		24·9	86·4		34·4	66·1		24·4	50·9		16·7
Median no. of RUTF		51			79			59			46	
IQR		42–68			63–104			49–76			40–57	
Consumption (kcal/kg per d)*	106		26·8	131·1		33·3	112		26·5	98·8		21·3

* To convert kcal to kJ, multiply by 4·184.

UNICEF Burkina Faso plans an average of 140 sachets per SAM child treated. The comparable group in OptiMA is the 1778 children who met standard SAM admission criteria and whose average RUTF ration was reduced by nearly half at 72·2 sachets per child treated. The 3072 children treated under OptiMA who fit the current MAM case definition consumed an average of 54·3 RUTF sachets/child which is lower than the 60–90 ready-to-use supplementary food sachets/child typically planned for MAM programming.

Table 6 shows factors associated with recovery after controlling for age and sex: alive mother status (adjusted hazard ratio 1·39; 95 % CI 1·02, 1·89), child of a caretaker who received MUAC training (adjusted hazard ratio 1·09; 95 % CI 1·01, 1·19) and who lived close to health centres (adjusted hazard ratio 1·25; 95 % CI 1·11, 1·42) were more likely to recover. Conversely, children who did not receive seasonal malaria chemoprevention during July and October were less likely to recover (0·87; 95 % CI 0·81, 0·94).

**Table 6.** Factors associated and attributable fraction (AF) with nutritional recovery (*n* 4163) among all children (*n* 4958) included in Optimising treatment for acute MAlnutrition (OptiMA) protocol, Yako district, Burkina Faso, 2017* (Numbers and percentages; hazard ratios (HR) and 95 % confidence intervals)

	*n*/*N*	%	Univariate analysis			Multivariate analysis (*n* 4201)			AF	
			Crude HR	95 % CI	*P*	Adjusted HR	95 % CI	*P*	AF	95 % CI
Child socio-demographic characteristics										
Age category (*n* 4958)										
<24 months	3376/4077	82·8	1			1		<0·0001		
≥24 months	787/881	89·3	1·61	1·48, 1·75		1·61	1·48, 1·75		9·7	8·4, 13·1
Sex (*n* 4941)										
Female	2381/2884	82·6	1		<0·0001	1		<0·0001		
Male	1768/2057	86	1·21	1·14, 1·29		1·21	1·13, 1·29		8·1	4·0, 10·4
Mode of referral (*n* 4845)										
Outpatient consultation	1869/2253	83	1		0·05					
Community agent	1619/1904	85	1·04	0·97, 1·11						
Mothers screening	545/628	86·8	0·9	0·81, 1·01						
Inpatient consultation	49/60	81·7	1·15	0·86 , 1·53						
Distance to the health centre (km) (*n* 4703)										
≥10	376/447	84·1	1		<0·0001	1		<0·001		
<10	3585/4256	84·2	1·28	1·13, 1·45		1·25	1·11, 1·42		18·1	11·0, 24·8
Mother’s characteristics at admission										
Mother’s status (*n* 4873)										
Death	53/67	79·1	1		0·04	1		0·03		
Alive	4047/4806	84·2	1·36	1·02, 1·81		1·39	1·02, 1·89		26·9	9·5, 40·8
No. of siblings (*n* 4697)										
0	13/18	72·2	1		0·9					
1–4	2267/2711	83·6	0·97	0·57, 1·63						
4–12	1683/1968	85·5	0·98	0·58, 1·66						
Breast-feeding (*n* 4512)										
No	597/692	86·3	1		<0·0001					
Yes	3226/3820	84·5	0·76	0·70, 0·83						
MUAC training										
No	881/1111	79·3	1		0·02	1		0·05		
Yes	3201/3733	85·7	1·10	1·01, 1·20		1·09	1·0, 1·19		6·7	1·8, 12·0
Malaria prevention										
At least one RDT positive										
Yes	1160/1354	85·7	1		0·2					
No	2916/3504	83·2	1·05	0·98, 1·13						
SMC† at admission										
Included out of SMC campaign‡	2998/3350	84·5	1		0·002	1		0·001		
SMC campaign‡ eligible and received	258/305	84·6	1·09	0·96, 1·25		1·06	0·93, 1·22		6·6‡	2·5, 12·7
SMC campaign‡ eligible and not received	872/1052	82·9	0·89	0·82, 0·96		0·87	0·81, 0·94			

MUAC, mid upper arm circumference; RDT, rapid diagnostic tests; SMC, seasonal malaria chemoprevention.

* Shared frailty model with a random effect on health centres censoring at 12 weeks of inclusion in the programme.

† SMC campaign period from July to October 2017.

‡ SMC at admission was combined in two categories ‘included out of SMC campaign’ and ‘included during SMC campaign’, the reference chosen for the AF calculation was ‘included during SMC campaign’.

Globally, AF indicate that 57·1 % (95 % CI 46·5, 69·6) of the recovery was explained by the six variables selected in the final model. The two variables that explain most of the recovery are: children whose mother is alive (AF 28·9 % (95 % CI 9·5, 40·8) and living within 10 km of the health centre (18·1 %, 95 % CI 11·0, 24·8).

## Discussion

This proof-of-concept trial evaluated a therapeutic nutrition protocol for children affected by acute malnutrition, defined as MUAC < 125 mm or oedema, and treated with one product (RUTF) at a gradually reduced dose based on a child’s weight and MUAC status. The OptiMA protocol was implemented in a ‘real-life’ setting by Burkinabe Ministry of Health personnel in all fifty-four health centres of Yako District, with operational support from a national and international non-governmental consortium and an established UNICEF-supported RUTF supply chain. Nurses had minimal difficulty adapting to this new protocol as evidenced by excellent adherence to the new dosing table and application of the new admission criteria. Programme outcomes overall were satisfactory with substantial reduction in RUTF ration compared with standard SAM treatment. However, recovery and non-response rates were unsatisfactory in children admitted at MUAC < 115 mm or oedema.

Overall recovery and mortality rates reported here, 86·3 and 0·4 % respectively, compare favourably to the 83 % recovery and 4 % mortality reported by a similar MUAC-based study in Sierra Leone, which had a more rapid reduction in RUTF dosage than OptiMA^(11)^. A MUAC-based programme previously implemented in the same district of Burkina Faso and using MUAC < 120 mm for admissions reported 91·6 % recovery and 1·1 % mortality, but the present study did not use a reduced RUTF regimen and had a less stringent discharge criterion of a single measure of MUAC ≥ 124 mm^(6)^.

Recovery in the category of children admitted at MUAC < 115 mm or oedema (70·4 %) was poorer than anticipated; this was largely attributable to high non-response (10·6 %) and defaulter rates (9·3 %). Non-responders and defaulters did achieve an average MUAC of 119·5 and 115·1 mm at exit, respectively, while the average MUAC at admission in the MUAC < 115 mm group was 109·5 mm. Mortality remained low at 1·5 % and is similar to the mortality rate of 1·6 % reported for children with MUAC < 115 mm at admission in the prior MUAC-based programme in this same district^(6)^. Elsewhere among MUAC-based programmes without RUTF reduction, recovery rates for this category have ranged from 57·4 % in India, 63·4 % in Malawi and 82 % in Sudan^(20–22)^. In the MUAC-based programme with RUTF dose reduction in Sierra Leone recovery for this sub-group was 75·3 %^(23)^; however, nutritional oedema was more prevalent in Sierra Leone (2·9 *v.* 0·7 % in OptiMA). This range of reported recovery rates with and without RUTF dose reduction suggests that recovery for this category is challenging. Other important factors like underlying disease (HIV, tuberculosis, sickle cell disease) might have contributed to a low recovery rate.

Similarly, recovery rate was the lowest (64·3 %) for children admitted with MUAC < 115 mm and WHZ < –3 (8·8 % of all admissions) and is again driven primarily by a high non-response rate at 12·5 %. Stunting was also highly prevalent in this group, reaching 57·9 %. Previous studies described a correlation between shorter length and treatment non-response rate, suggesting that severely wasted and stunted children may require other interventions over a longer time period to improve growth trajectory^(7,24)^.

The sub-optimal outcomes for the small proportion of severely wasted and stunted children should be interpreted in light of overall programme performance. Under OptiMA, only 16 % of children treated were admitted with MUAC < 115 mm or oedema, while 84 % were admitted with MUAC between 115 and 124 mm, whereas the proportion of children in each category in the Sierra Leone study was 30 and 70 %^(11)^. This might suggest that the OptiMA programme achieved good coverage and caught most children early in the wasting process. Thus, those children who exhibit severe wasting and stunting, in spite of apparently good access to treatment, may represent either a history of low birth weight or malnutrition secondary to chronic illness, both of which lower expectations for treatment outcomes.

Current SAM programmes typically plan RUTF consumption between 120 and 150 sachets per child treated, while MAM programmes plan between sixty and ninety sachets of ready-to-use supplementary food per child^(25,26)^. However, comparisons of average rations must consider variations in discharge criteria. Under OptiMA, discharge was stringent: two consecutives measures of MUAC ≥ 125 mm. In this light, the overall RUTF consumption of 60·8 sachets average per child per course of treatment is highly encouraging. However, further study will be needed to determine if increasing RUTF dosage for children admitted with MUAC < 115 mm will improve recovery and non-response even if other factors appear to influence recovery rate as shown in the multivariate analysis.

Female sex bias in MUAC-based nutrition programmes has been documented in previous studies^(11,27)^, similar to what the present study observed with 58·4 % of girls at admission. Isanaka *et al*. also clearly show that WHZ score preferentially selects males^(27)^. Thus combining anthropometric criteria, MUAC and WHZ serve to maintain sex balance in nutrition programmes. Using MUAC as the single anthropometric criterion for programme admission may require improving MUAC sensitivity for boys and specificity for girls.

The multivariable survival model highlighted that recovery was positively associated with male children older than 24 m, living within 10 km of the health facility, whose mother was alive and had been trained to use MUAC. Recovery was negatively associated with children who had not received seasonal malaria chemoprevention between July and October. Most of these results have already been found in other studies. It is well known that female and younger children present lower MUAC^(28)^. As for distance to the health facility, a study from Ethiopia showed that SAM children living within 25 min of the facility were 1·53 times more likely to recover^(29)^. It has also been shown that mothers screening their children by MUAC can lead to earlier initiation of care^(12)^. The low proportion of self-referral by mothers (12·9 %) is likely due to an underestimation because many mothers sought confirmation from a Community Health Worker (CHW) after using MUAC at home and then reported being referred by a CHW at admission. To the best of our knowledge, it is the first time that a positive association is demonstrated between training mother to use MUAC bracelet and recovery, likely due to a better care-seeking behaviour resulting from such trainings. The negative association between children who did not receive CPS should be viewed with caution because the reason why these children did not receive the seasonal malaria chemoprevention was unknown and we cannot exclude a confusion bias or even reverse causality. Of these six variables associated with recovery, AF calculation highlighted, unsurprisingly, that recovery was primarily driven by maternal vital status and living within 10 km of the health facility.

The main limitation in the present study is the absence of a comparator group. In order to determine how MUAC-based integrated SAM and MAM protocols perform compared with current protocols that rely on MUAC and WHZ criteria, additional randomised trials, in multiple contexts, are necessary. Another limitation is that we did not collect data on possible RUTF sharing. But, it was previously shown in a study on MAM treatment in the same location that nearly two-thirds of children consumed all of the prescribed LNS ration themselves^(30)^.

Lastly, this analysis relied on routinely collected programme data. Extensive data monitoring was carried out throughout the study to ensure that all children found in health centre registers were retrieved in the OptiMA database. To our knowledge, such monitoring is rarely performed in programmatic studies. The strength of the present study is that it shows it is possible to conduct robust data monitoring in a ‘real-life’ programme to generate a high-quality individualised database. However, it was not without challenge. The reclassification of children erroneously categorised at exit-influenced OptiMA’s recovery rate downwards. Adherence to routine amoxicillin prescription for children with MUAC < 120 mm or oedema as specified in the protocol was uneven and thus precluded any analysis of correlation between this prescription and recovery.

### Conclusion

A simplified, combined SAM and MAM protocol based on early detection through household MUAC screenings, treatment with one product determined by the presence of oedema and/or MUAC < 125 mm, and progressive RUTF dose reduction had programme outcomes that exceeded national Burkina Faso and international SPHERE standards. This early detection and treatment initiation with progressively reduced RUTF ration shows promise and warrants further operational pilots in different contexts. However, randomised control trials are needed to compare the strategy to current national protocols^(31)^. Because of low recovery among children admitted with MUAC < 115 mm, further study is also needed to determine whether increasing RUTF dosage for the most severely malnourished will improve outcomes.

## Supplementary material

For supplementary material accompanying this paper visit http://dx.doi.org/10.1017/S0007114519003258. click here to view supplementary material
