# Supplementary material for: New approach to simplifying and optimising acute malnutrition treatment in children aged 6–59 months: the OptiMA single-arm proof-of-concept trial in Burkina Faso
Source: Br J Nutr. 2019 Dec 10;123(7):756–67. doi: 10.1017/S0007114519003258 (PMC7054246; doi:10.1017/S0007114519003258)
Supplement: Supplementary file 1 [file S0007114519003258sup001.docx]

**Supplementary File #1**

Description of children included in “Non-MUAC eligible” arm and OptiMA protocol. Yako district, Burkina Faso, 2017.

|  |  | **Non MUAC eligible** | | | **OptiMA protocol** | |
| --- | --- | --- | --- | --- | --- | --- |
|  |  | | **N=117** | | **N=4958** | |
|  |  | | **n** | **%** | **n** | **%** |
| **Age** (*month, mean (sd*)) |  | | 23.1 | (12.1) | 14.9 | 8.8 |
|  |  | |  |  |  |  |
| **Age category** | <24 m | | 72 | 61.5 | 4077 | 82.2 |
|  | > 24 m | | 45 | 38.5 | 881 | 17.8 |
|  |  | |  |  |  |  |
| **Sex** | M | | 74 | 63.2 | 2057 | 41.6 |
|  | F | | 43 | 36.8 | 2884 | 58.4 |
|  |  | |  |  |  |  |
| **Hospitalization** |  | | 13 | 11.1 | 680 | 13.7 |
|  |  | |  |  |  |  |
| **Exit status** | Recovered | | 111 | 94.9 | 4279 | 86.3 |
|  | Defaulted | | 3 | 2.6 | 233 | 4.7 |
|  | Deceased | | 2 | 1.7 | 22 | 0.4 |
|  | Not respondent | | - | - | 170 | 3.4 |
|  | Transfered | | 1 | 0.8 | 11 | 0.2 |
|  | Alive, unconfirmed status | | - | - | 243 | 4.9 |
